# Time‐Restricted Access to High‐Fat Diet Influences Weight Gain, Meal Patterns, and Food Preference

**DOI:** 10.1002/oby.70030

**Published:** 2025-09-25

**Authors:** Payam A. Fathi, Michelle B. Bales, Pranav Sathu, Julio E. Ayala

**Affiliations:** ^1^ Department of Molecular Physiology & Biophysics Vanderbilt University School of Medicine Nashville Tennessee USA; ^2^ Vanderbilt Mouse Metabolic Phenotyping Center‐Live Nashville Tennessee USA; ^3^ Vanderbilt Center for Addiction Research Nashville Tennessee USA

**Keywords:** diet preference, meal patterns, time‐restricted high‐fat diet

## Abstract

**Objective:**

Access to only high‐fat diet (HFD) during the light versus dark cycle promotes different metabolic outcomes. We assessed changes in body weight/composition, feeding behavior, and metabolic parameters in mice fed HFD during the light or dark cycle with concomitant ad libitum access to chow.

**Methods:**

Male C57BL/6J mice were housed in metabolic chambers with two hoppers containing chow. HFD was then provided in one hopper, with access restricted to the light or dark cycle. The other hopper provided ad libitum access to chow. Food intake, meal patterns, energy expenditure, activity, and substrate oxidation were measured for ~4 weeks. Body weight/composition was measured before and after ~4‐week HFD access.

**Results:**

Light cycle HFD access promoted greater weight and fat mass gain. Although daily caloric intake was equivalent between groups, light cycle HFD access increased preference for HFD and intake of larger, more frequent HFD meals during the daytime. Dark cycle HFD access promoted preference for chow and consumption of larger, more frequent chow meals.

**Conclusions:**

Light cycle HFD access parallels detrimental metabolic outcomes of ad libitum HFD access. Dark cycle HFD access reduces weight gain and adiposity; this is associated with enhanced chow preference.

**Study Importance**

- What is already known?
  ○ Providing ad libitum access to high‐fat diets increases daytime caloric intake and promotes obesity.
  ○ Restricting access to high‐fat diets during the daytime versus nighttime in mice promotes different effects on weight gain and metabolic disorders.
- What does this study add?
  ○ Restricting a high‐fat diet to the light cycle with concomitant access to a chow diet promotes greater weight gain and adiposity compared to providing access to a high‐fat diet during the dark cycle along with ad libitum access to chow.
  ○ Access to a high‐fat diet and chow during the day increases the preference for the high‐fat diet and high‐fat meal size and frequency whereas access to both a high‐fat diet and chow during the nighttime increases the preference, meal size, and frequency from the chow diet.
- How might these results change the direction of research or the focus of clinical practice?
  ○ Our findings suggest that time‐restricted access to high‐fat diets promotes vastly different effects on feeding behavior, mainly food preference and meal patterns, that parallel behaviors observed in humans with dysregulated sleep/wake cycles (e.g., night shift workers), a population that is at increased risk of developing obesity.

## 1 Introduction

Increased consumption of calorie‐dense foods and beverages is a significant contributor to weight gain and obesity [1]. Rodent studies typically use ad libitum access to a high‐fat diet (HFD) to promote obesity. However, alterations to behavior resulting from this approach do not closely mimic the human condition. When provided ad libitum access to HFD, mice increase caloric intake during their inactive period (light cycle) [2, 3]. This disrupts not only feeding behavior, such as meal patterns, but also circadian gene expression and circulating metabolite patterns [2, 3, 4].

Providing mice with an HFD only during the active period (dark cycle) prevents excessive weight gain and metabolic dysregulation, while providing HFD access only during the inactive period promotes weight gain and metabolic dysregulation independently of total caloric intake [5, 6, 7]. The latter is akin to the increased risk of weight gain and metabolic dysregulation observed in human night shift workers [8, 9]. Thus, the timing of HFD exposure is an important factor in the development of obesity.

One caveat to interpreting studies providing mice cycle‐dependent access to HFD is that mice had access to *only* HFD during these periods. To more closely recreate diet choice, the present studies investigated behavioral and metabolic effects in mice fed HFD only during the light or dark cycle concomitant with ad libitum exposure to chow. We demonstrate that mice given access to HFD during the light cycle gain more weight and fat mass than mice given access to HFD during the dark cycle, yet daily caloric intake is equivalent in both groups. Mice given access to HFD during the light cycle prefer the HFD, but mice given access to HFD during the dark cycle prefer the chow diet. Differences in meal patterns also emerge in mice given access to HFD during the light versus dark cycle that could influence weight gain. These studies suggest that the timing of exposure to obesogenic diets not only modulates metabolic outcomes (e.g., weight and fat mass gain) but also food preference and other feeding behaviors that could ultimately contribute to these metabolic outcomes.

## 2 Methods

### 2.1 Animals

Six‐week‐old male C57Bl6/J mice (purchased from The Jackson Labs) were fed chow (2.86 kcal/g; 5001—PicoLab Rodent Diet) and were studied at 10–12 weeks of age. Mice were maintained on a 12–12 light–dark cycle (0600‐1800) at ~23°C. Procedures were approved by the Institutional Animal Care and Use Committee at Vanderbilt University.

### 2.2 Diet Access Experiments

**FIGURE 1:**
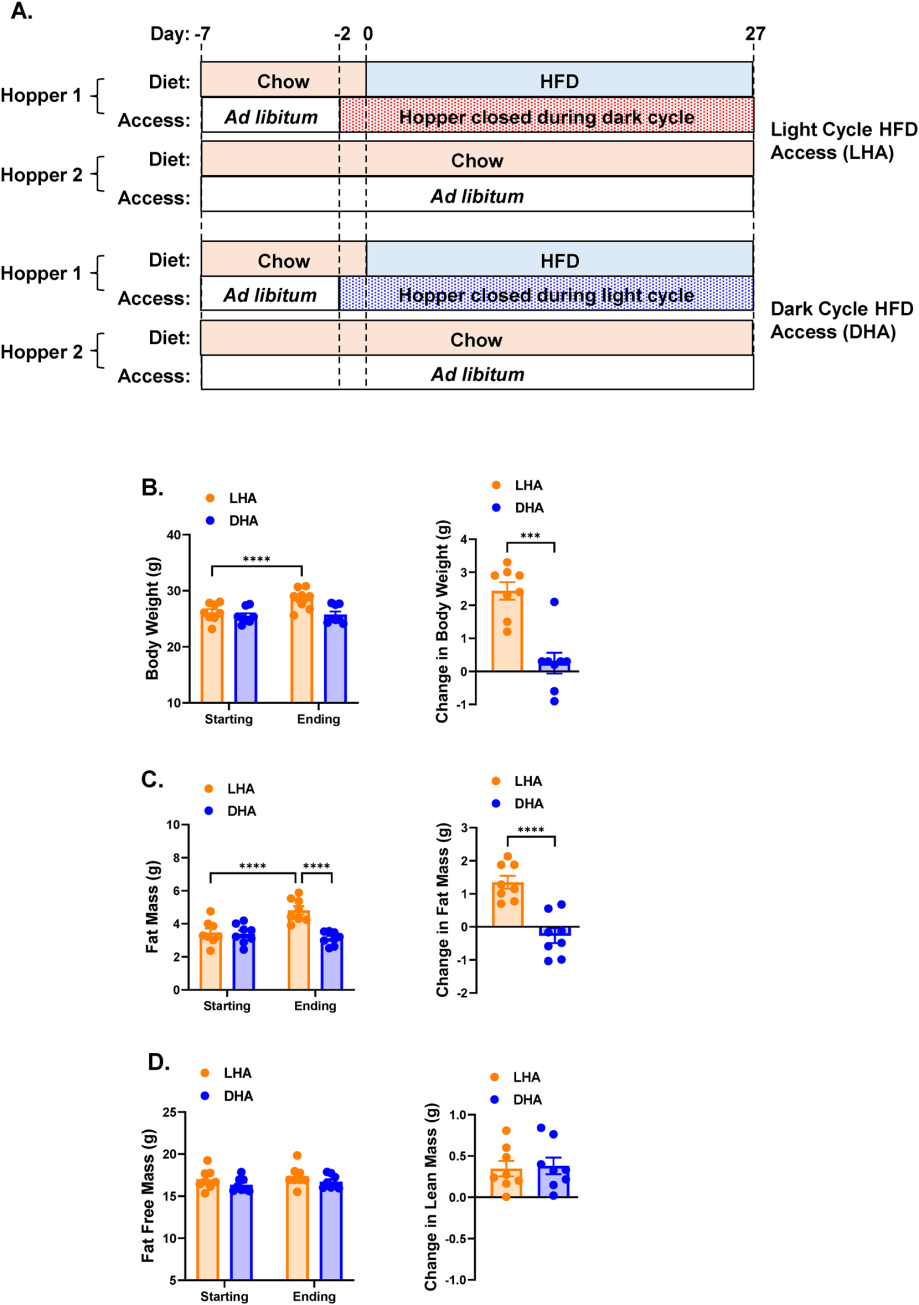
Study design, body weight, and body composition in mice given access to HFD during the light (LHA) versus dark (DHA) cycle. (A) Study design: Chow‐fed mice were placed in metabolic chambers to acclimate for 7 days. The chow diet was accessible from two hoppers (Hoppers 1 and 2). Over the last 2 days of acclimation, Hopper 1 was closed for half the mice during the light cycle and the other half during the dark cycle. On Day 0, the chow diet in Hopper 1 was replaced with HFD, and this hopper continued to be closed during the same cycle it was previously closed. Hopper 2 contained chow and remained open. This continued for 27 days. (B) Absolute body weight on Day 0 (Starting) and Day 27 (Ending) and change in body weight from Day 0 to 27. (C) Absolute fat mass on Day 0 (Starting) and Day 27 (Ending) and change in fat mass from Day 0 to 27. (D) Absolute fat free mass on Day 0 (Starting) and Day 27 (Ending) and change in fat free mass from Day 0 to 27. Data are shown as mean ± SEM for *N* = 8 per group and were statistically analyzed using a *t*‐test or mixed model ANOVA with Tukey's multiple comparison tests. *p* value definitions: ***< 0.001, ****< 0.00001. [Color figure can be viewed at wileyonlinelibrary.com]

Sixteen mice were single housed in Promethion Metabolic chambers (Sable Systems International) with ad libitum access to chow in two food hoppers (Hopper 1 and Hopper 2) for a 7‐day acclimation and chow data acquisition period (Figure 1A). Mice were randomly assigned to one of two groups: Light Cycle HFD Access (LHA) and Dark Cycle HFD Access (DHA). Each group consisted of eight mice. During the last 2 days of the chow period, a computer‐controlled access panel was closed for Hopper 1 during the light cycle (0600‐1800) for the mice assigned to the DHA group and for Hopper 1 during the dark cycle (1800‐0600) for the mice assigned to the LHA group. On day 0, Hopper 1 was filled with 60% HFD (5.24 kcal/g; Research Diets Inc., D12492). Access to this HFD‐containing hopper was restricted to the light cycle in LHA mice or the dark cycle in DHA mice. Hopper 2 contained chow and remained continuously accessible. Mice remained in the metabolic chambers for 27 days. The location of Hoppers 1 and 2 was switched every 4 days to minimize location bias and association of a specific hopper location with a given diet. Fresh HFD was also given at these times.

### 2.3 Metabolic Measurements

Food intake, locomotor activity, respiratory exchange ratio (RER), and energy expenditure (EE) were continuously measured across 5‐min intervals in the Promethion metabolic chambers. Energy intake was calculated by multiplying mass consumed by the diet caloric density. EE was calculated using the Weir equation [10]; EE (kcal/h) = ([3.941·VO_2_] + [1.106·VCO_2_])·0.06, where VO_2_ is the rate of oxygen consumption and VCO_2_ is the rate of carbon dioxide production measured by gas exchange in the metabolic chambers. Energy balance was calculated by subtracting EE from energy intake. Activity was estimated by the number of infrared beam breaks in the *X* and *Y* plane and multiplied by the distance between beam breaks. Body weight and body composition (NMR, Bruker Optics) were measured at Day 0 (last day of chow only exposure) and Day 27 of the timed HFD period.

### 2.4 Meal Parameters

A meal was defined as food removal spanning at least 30 s, a minimum of 10 mg to account for inherent sensor limitations, a maximum of 500 mg to account for spillage, and a threshold of 5 min for intermeal intervals based on prior rodent literature [11, 12]. Satiety index was calculated as the ratio of intermeal interval and meal size. HFD was packed in the hopper to reduce spillage. Cages were inspected daily for excessive spillage (food on cage bottom) and noted. If total food intake and meal number for a given day were greater than 1.5× standard deviation of the mean for its respective feeding period, then this was considered excessive spillage, and intake and meal pattern data for that day were excluded.

### 2.5 Statistical Analysis

Raw data were processed using the Sable Systems Macro Interpreter (version 23.6). Graphs were generated and data statistically analyzed using, as appropriate and indicated in the figure legend, t‐test, one‐way ANOVA, or mixed model ANOVA with Tukey's post hoc for multiple comparisons using GraphPad Prism (version 10). Significance was set at *p* < 0.05.

## 3 Results

### 3.1 Changes in Body Weight and Body Composition in Mice Given Access to HFD During the Light Versus the Dark Cycle

All mice had similar starting body weights prior to being given access to HFD (Figure 1B). Following ~4 weeks of HFD exposure, LHA mice had higher body weight and weight gain than DHA mice (Figure 1B) due to a greater increase in fat mass (Figure 1C). There was no difference in fat free mass between groups (Figure 1D).

### 3.2 Daily Food and Energy Intake in Mice With Access to HFD During the Light Versus Dark Cycle

**FIGURE 2:**
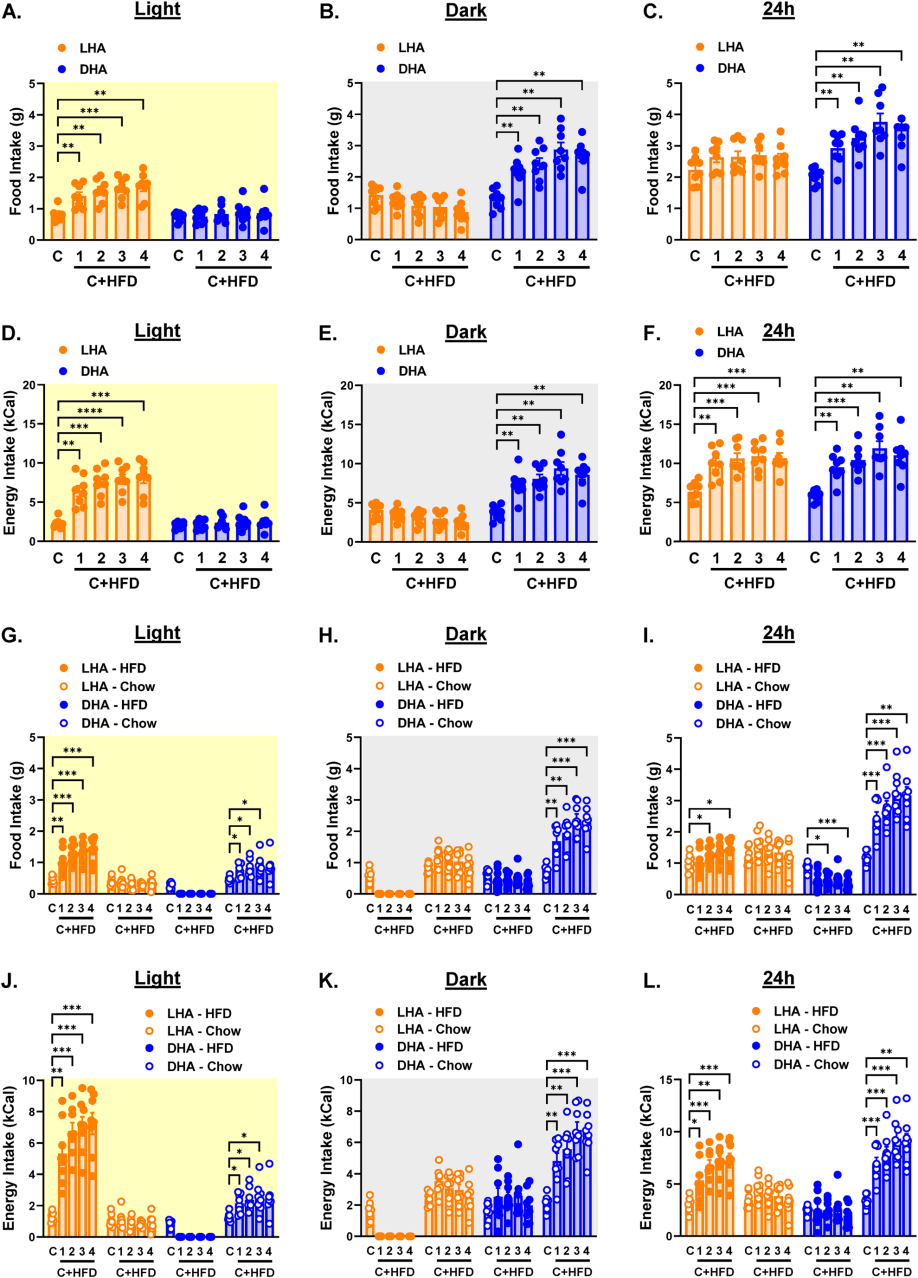
Food and energy intake in light cycle HFD access (LHA) and dark cycle HFD access (DHA) mice. Light cycle data are shaded yellow, dark cycle data are shaded gray, and 24‐h data are shaded white. Average weekly food intake during the (A) light cycle, (B) dark cycle, and (C) 24 h. Average weekly energy intake during the (D) light cycle, (E) dark cycle, and (F) 24 h. Average weekly food intake by diet during the (G) light cycle, (H) dark cycle, and (I) 24 h. Average weekly energy intake by diet during the (J) light cycle, (K) dark cycle, and (L) 24 h. C = chow week; 1, 2, 3, 4 C + HFD = weeks 1, 2, 3, and 4 of access to both chow and HFD. Data are shown as mean ± SEM for *N* = 8 per group and were statistically analyzed using mixed model ANOVA with Tukey's multiple comparison tests. *p* value definitions: *< 0.05, **< 0.01, ***< 0.001, ****< 0.00001. Although not shown, differences with time points during which hoppers were closed were significant at *p* < 0.00001. [Color figure can be viewed at wileyonlinelibrary.com]

Ad libitum exposure to HFD increases food and energy intake, so we investigated whether cycle‐restricted access to HFD results in similar effects. For all data, weekly averages for the chow only week (C) and weeks 1–4 of chow+HFD (C + HFD) are shown in the main figures, and daily averages are shown in online Supporting Information: Figures. Food intake increased during the cycle in which the HFD was available (Figure 2A,B and Figure S1A,B). DHA mice displayed a greater increase in daily (24 h) food intake than LHA mice (Figure 2C and Figure S1C). Total energy intake increased during the cycle in which the HFD was available (Figure 2D,E and Figure S1D,E). However, the total daily energy intake increased equally in LHA versus DHA mice (Figure 2F and Figure S1F).

The observation that DHA mice have higher daily food intake but equivalent daily energy intake compared to LHA mice suggests a difference in the relative amount of chow versus HFD consumed. We assessed the mass and energy intake of chow and HFD separately. LHA mice increased HFD intake by mass and energy while chow intake remained unchanged during the light cycle (Figure 2G,J and Figure S1G,J). Surprisingly, dark cycle HFD intake by mass and energy did not increase in DHA mice (Figure 2H,K and Figure S1H,K). Instead, both light and dark cycle chow intake increased by mass and energy in DHA mice (Figure 2G,H,J,K and Figure S1G,H,J,K). Thus, daily food intake in LHA mice was made up of increased mass and energy intake from the HFD (Figure 2I,L and Figure S1I,L). Conversely, DHA mice displayed a significant increase in daily food and energy intake from chow (Figure 2I,L and Figure S1I,L). This relative distribution was observed in the total energy intake of chow versus HFD over the ~4‐week HFD exposure period, even as total energy intake was not different between LHA and DHA mice (Figure S1M).

### 3.3 Changes in Energy Expenditure, Activity, Energy Balance, and Substrate Oxidation in Mice With Access to HFD During the Light Versus Dark Cycle

**FIGURE 3:**
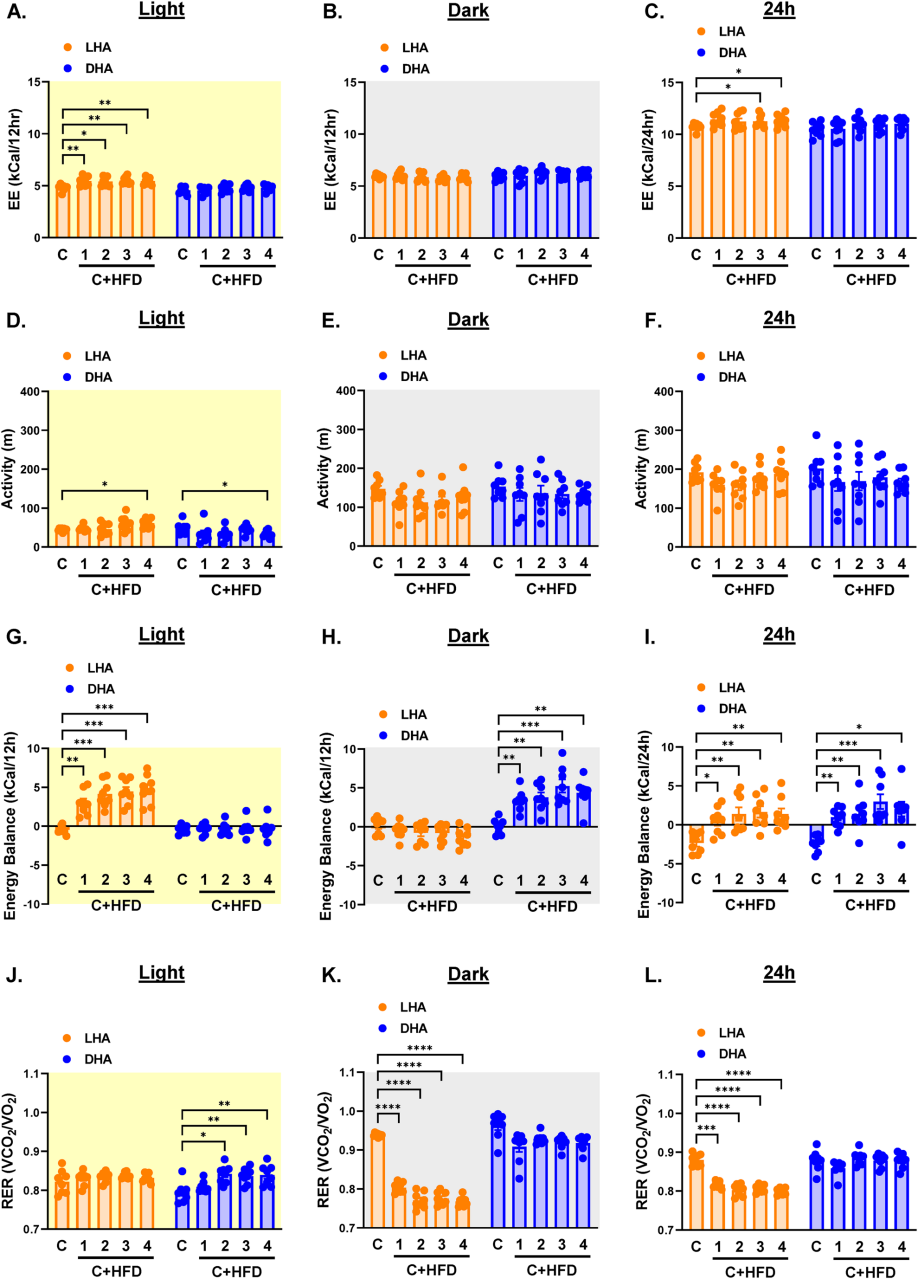
EE, activity, energy balance, and RER in light cycle HFD access (LHA) and dark cycle HFD access (DHA) mice. Light cycle data are shaded yellow, dark cycle data are shaded gray, and 24‐h data are shaded white. Average weekly EE during the (A) light cycle, (B) dark cycle, and (C) 24 h. Average weekly locomotor activity during the (D) light cycle, (E) dark cycle, and (F) 24 h. Average weekly energy balance during the (G) light cycle, (H) dark cycle, and (I) 24 h. Average weekly RER during the (J) light cycle, (K) dark cycle, and (L) 24 h. C = chow week; 1, 2, 3, 4 C + HFD = weeks 1, 2, 3, and 4 of access to both chow and HFD. Data are shown as mean ± SEM for *N* = 8 per group and were statistically analyzed using mixed model ANOVA with Tukey's multiple comparison tests. *p* value definitions: *< 0.05, **< 0.01, ***< 0.001, ****< 0.00001. [Color figure can be viewed at wileyonlinelibrary.com]

Our previous studies showed that exposure to HFD rapidly increases EE [3]. This was observed during the light cycle in LHA mice (Figure 3A and Figure S2A). Contrasting this, EE was unaffected during either cycle in DHA mice (Figure 3A,B and Figure S2A,B), resulting in a slight increase in daily EE only in LHA mice (Figure 3C and Figure S2C). Light cycle activity slightly increased in LHA mice but slightly decreased in DHA mice, and dark cycle activity remained unchanged in both groups (Figure 3D,E and Figure S2D,E). Mice displayed a positive energy balance corresponding with the cycle in which the HFD was available (Figure 3G,H and Figure S2G,H). Daily energy balance also became more positive in both groups (Figure 3I and Figure S2I).

Differences in dietary preference between LHA and DHA mice (Figure 2G–L) are reflected in RER. Regardless of the cycle of HFD exposure, RER remained at ~0.8 during the light cycle, although light cycle RER increased slightly in DHA mice (Figure 3J and Figure S2J). This is indicative of higher fat oxidation relative to carbohydrate oxidation. Dark cycle RER significantly dropped in LHA mice (Figure 3K and Figure S2K), likely reflecting an increased reliance on fat stores accumulated during the daytime. Contrasting this, dark cycle RER remained elevated in DHA mice (Figure 3K and Figure S2K) due to their nighttime increase in chow intake. Daily RER decreased in LHA mice but remained unchanged in DHA mice (Figure 3L and Figure S2L).

### 3.4 Meal Patterns in Response to Cycle‐Specific HFD Exposure

**FIGURE 4:**
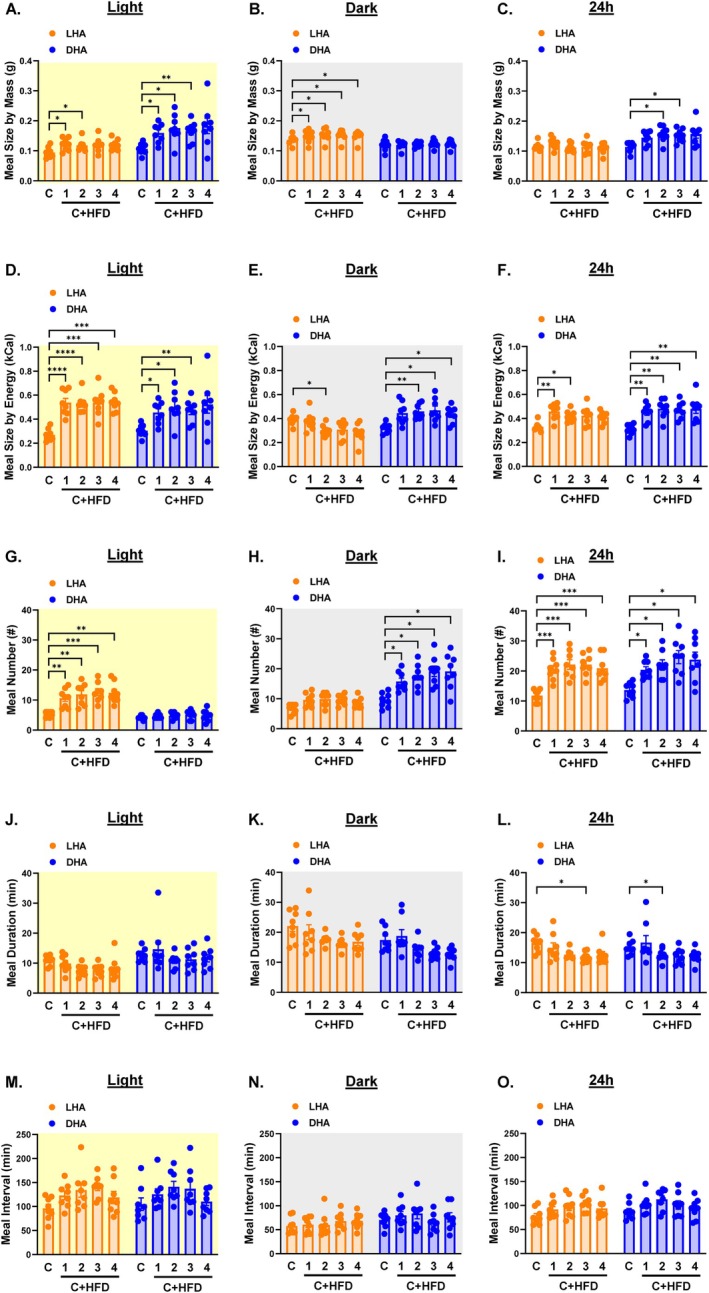
Meal patterns in light cycle HFD access (LHA) and dark cycle HFD access (DHA) mice. Light cycle data are shaded yellow, dark cycle data are shaded gray, and 24‐h data are shaded white. Average weekly meal size by mass during the (A) light cycle, (B) dark cycle, and (C) 24 h. Average weekly meal size by energy content during the (D) light cycle, (E) dark cycle, and (F) 24 h. Total weekly meal number during the (G) light cycle, (H) dark cycle, and (I) 24 h. Average weekly meal duration during the (J) light cycle, (K) dark cycle, and (L) 24 h. Average weekly intermeal interval during the (M) light cycle, (N) dark cycle, and (O) 24 h. C = chow week; 1, 2, 3, 4 C + HFD = weeks 1, 2, 3, and 4 of access to both chow and HFD. Data are shown as mean ± SEM for *N* = 8 per group and were statistically analyzed using mixed model ANOVA with Tukey's multiple comparison tests. *p* value definitions: *< 0.05, **< 0.01, ***< 0.001, ****< 0.00001. [Color figure can be viewed at wileyonlinelibrary.com]

We next investigated the effect of cycle‐dependent HFD exposure on meal patterns. LHA mice displayed a transient increase in light cycle meal size by mass (Figure 4A and Figure S3A) that translated to a significant increase in light cycle meal size by energy (Figure 4D and Figure S3D). Dark cycle meal size by mass also increased slightly in LHA mice (Figure 4B and Figure S3B), although this did not increase meal size by energy (Figure 4E and Figure S3E) since these mice only had access to chow during the dark cycle. Unexpectedly, light cycle meal size increased by both mass and energy in DHA mice (Figure 4A,D and Figure S3A,D). Dark cycle meal size by mass was unaffected in these mice (Figure 4B and Figure S3B), but dark cycle meal size by energy increased (Figure 4E and Figure S3E). Average daily meal size by mass was unaffected in LHA mice but increased in DHA mice (Figure 4C and Figure S3C). This translated to a transient increase in daily meal size by energy in LHA mice and a sustained increase in meal size by energy in DHA mice (Figure 4F and Figure S3F).

Meal numbers increased in mice during the cycle when the HFD was accessible (Figure 4G,H and Figure S3G,H), resulting in increased daily meal numbers in both groups (Figure 4I and Figure S3I). There was a trend for meal duration to decrease during the light and dark cycles upon exposure to HFD in both groups (Figure 4J,K and Figure S3J,K). Thus, even though meal numbers increased, the reduced duration of each meal resulted in a trend toward increased intermeal interval (Figure 4M–O and Figure S3M–O).

**FIGURE 5:**
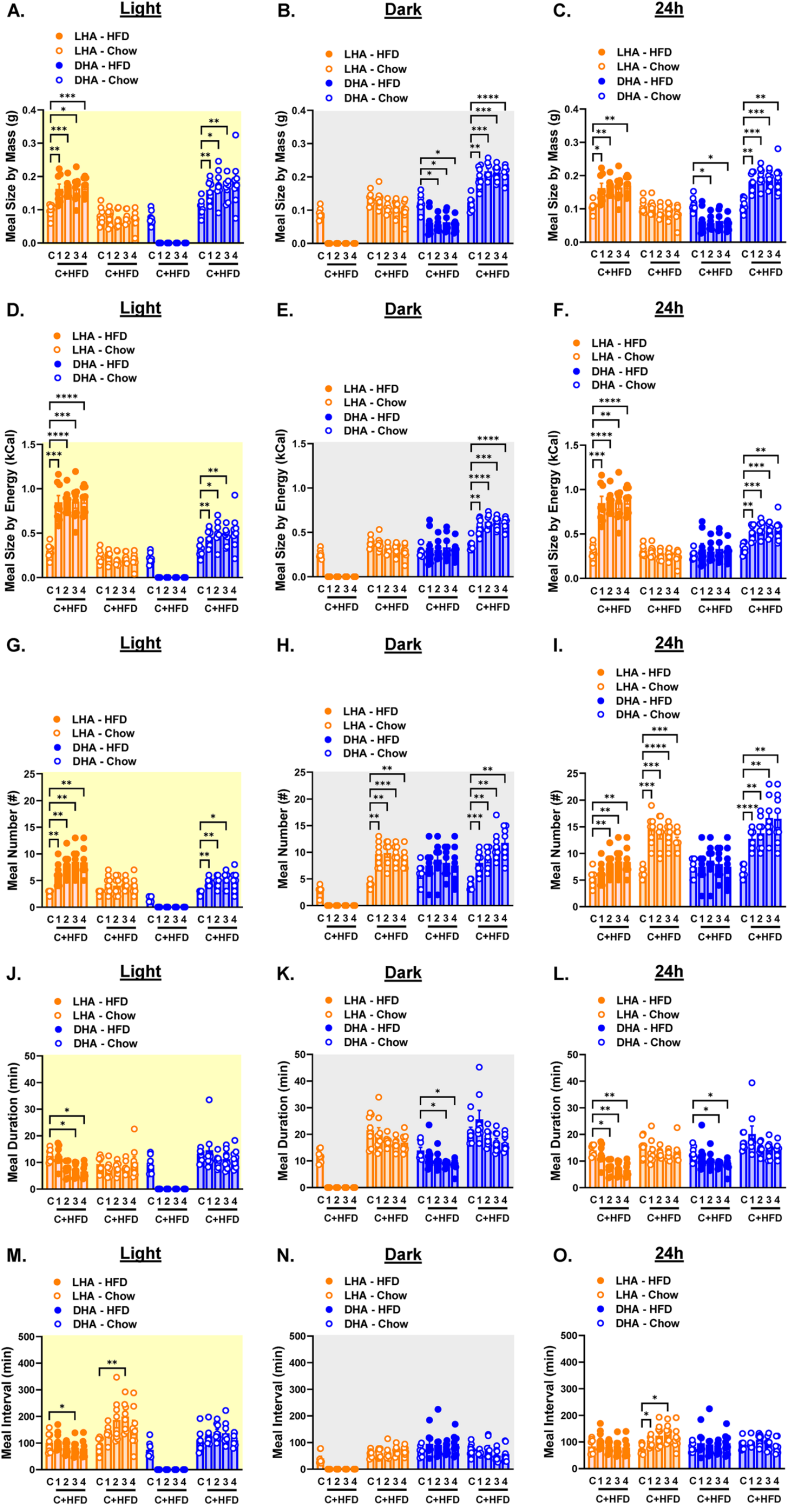
Meal patterns by diet in light cycle HFD access (LHA) and dark cycle HFD access (DHA) mice. Light cycle data are shaded yellow, dark cycle data are shaded gray, and 24‐h data are shaded white. Average weekly meal size by mass and by diet during the (A) light cycle, (B) dark cycle, and (C) 24 h. Average weekly meal size by energy content and by diet during the (D) light cycle, (E) dark cycle, and (F) 24 h. Total weekly meal number by diet during the (G) light cycle, (H) dark cycle, and (I) 24 h. Average weekly meal duration by diet during the (J) light cycle, (K) dark cycle, and (L) 24 h. Average weekly intermeal interval by diet during the (M) light cycle, (N) dark cycle, and (O) 24 h. C = chow week; 1, 2, 3, 4 C + HFD = weeks 1, 2, 3, and 4 of access to both chow and HFD. Data are shown as mean ± SEM for *N* = 8 per group and were statistically analyzed using mixed model ANOVA with Tukey's multiple comparison tests. *p* value definitions: *< 0.05, **< 0.01, ***< 0.001, ****< 0.00001. Although not shown, differences with time points during which hoppers were closed were significant at *p* < 0.00001. [Color figure can be viewed at wileyonlinelibrary.com]

The light cycle increase in meal size by mass and energy content in LHA mice was driven by an increase in the size of HFD meals (Figure 5A,D and Figure S4A,D), suggesting decreased HFD satiation. The mass and energy content of chow meals remained unchanged during both light and dark cycles in LHA mice (Figure 5A,D and Figure S4A,D). This translated to a significant increase in the daily mass and energy intake of HFD‐derived meals relative to chow‐derived meals in LHA mice (Figure 5C,F and Figure S4C,F). In DHA mice, dark cycle HFD meals were lower in mass compared to the mass of chow meals prior to the HFD switch, but the mass of chow meals increased during both the light and dark cycles (Figure 5A,B and Figure S4A,B). This is indicative of increased HFD satiation and relatively decreased chow satiation and resulted in the daily meal size by mass from the HFD being lower than that from the chow diet (Figure 5C and Figure S4C). Exposure to HFD during the dark cycle did not lead to higher energy content per meal from the HFD during either the dark cycle or 24‐h periods but instead increased the energy content of chow‐derived meals during the light, dark, and 24‐h cycles (Figure 5D–F and Figure S4D–F).

Exposure to HFD during the light cycle resulted in a greater number of daytime HFD‐derived meals with no change in chow‐derived meals (Figure 5G and Figure S4G). Interestingly, LHA mice increased the number of chow‐derived meals during the dark cycle (Figure 5H and Figure S4H). Similarly, the number of light and dark cycle meals from the chow diet increased in DHA mice (Figure 5G,H and Figure S4G,H). Surprisingly, in DHA mice, the number of dark cycle meals from the HFD did not increase relative to the number of chow meals prior to the diet switch (Figure 5H and Figure S4H). The total number of daily meals derived from the chow diet was higher than the number of meals from the HFD regardless of HFD cycle exposure (Figure 5I and Figure S4I), which is to be expected since the HFD was only available for 50% of the time. The tendency for light cycle and 24‐h meal duration to decrease in LHA mice was driven by reduced duration of HFD meals (Figure 5J,L and Figure S4J,L). Similarly, the tendency for dark cycle and 24‐h meals to be shorter in DHA mice was due to reduced duration of HFD meals (Figure 5K,L and Figure S4K,L). In LHA mice, a decreased interval between HFD meals was offset by an increased interval between chow meals during the daytime (Figure 5M and Figure S4M). There were no significant effects on intermeal intervals of either diet in DHA mice (Figure 5M–O and Figure S4M–O).

**FIGURE 6:**
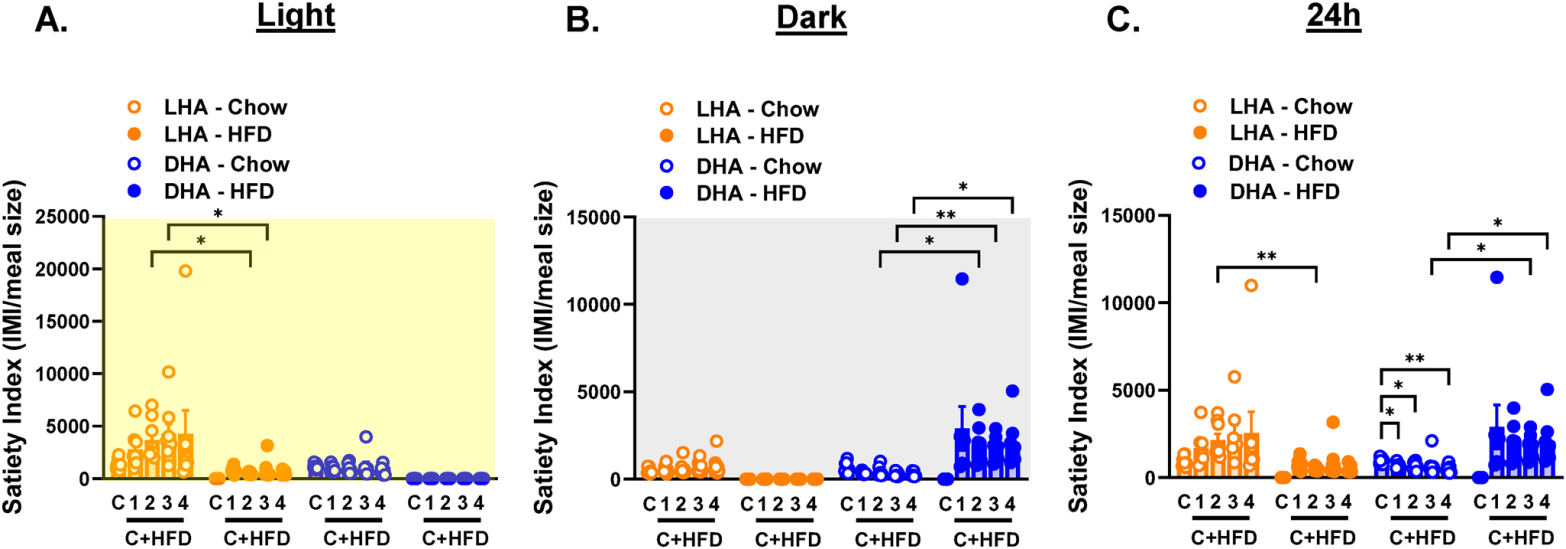
Satiety index in light cycle HFD access (LHA) and dark cycle HFD access (DHA) mice. Light cycle data are shaded yellow, dark cycle data are shaded gray, and 24‐h data are shaded white. Average weekly satiety index (intermeal interval (IMI)/meal size in grams) during the (A) light cycle, (B) dark cycle, and (C) 24 h. C = chow week. Data are shown as mean ± SEM for *N* = 8 per group and were statistically analyzed using mixed model ANOVA with Tukey's multiple comparison tests. *p* value definitions: *< 0.05, **< 0.01. [Color figure can be viewed at wileyonlinelibrary.com]

Based on these meal patterns, we calculated a satiety index as the ratio of intermeal interval to meal size by mass. Light cycle and 24‐h satiety was higher for the chow diet in LHA mice, and dark cycle and 24‐h satiety was higher for the HFD in DHA mice (Figure 6A–C). In sum, exposure to HFD during the light cycle promoted more frequent and larger HFD daytime meals of shorter duration but no dramatic effects on daytime chow meals. Conversely, exposure to HFD during the dark cycle led to smaller HFD meals of shorter duration and larger and more frequent chow meals.

## 4 Discussion

Consistent with previous studies [5, 6, 7], we demonstrate that mice gain greater weight and fat mass when given access to HFD during the inactive period versus the active period independently of total caloric intake. The novelty of the present studies is that low‐fat chow was also available with the HFD. This uncovered the unexpected finding that mice prefer chow over HFD only when the HFD is given at night. Furthermore, access to HFD during the light cycle results in more frequent and larger HFD meals of shorter duration, whereas exposure to HFD during the dark cycle leads to smaller HFD meals of shorter duration yet larger and more frequent chow meals. Thus, exposure to HFD during specific times of day influences food preference and meal patterns. This could provide insight into behaviors observed in humans with dysregulated sleep patterns, such as shift workers, who also display increased risk for developing obesity and other metabolic disorders [8].

Several studies suggest a relationship between circadian rhythms and metabolic phenotypes. Disruption of circadian clock genes promotes obesity and/or metabolic dysregulation [13, 14, 15]. Ad libitum access to HFD and obesity are associated with a loss of circadian gene expression in multiple tissues [4, 16, 17]. Restricting HFD access to the dark cycle resets the expression of circadian genes and protects mice from obesity and detrimental metabolic phenotypes [5, 7, 16]. The resetting of these circadian genes could orchestrate changes in feeding behavior that reduce excessive weight gain and protect from metabolic dysregulation. Alternatively, time‐restricted HFD exposure may improve feeding behavior via other mechanisms, and the resetting of circadian gene expression is secondary to improved metabolic outcomes. Future studies merit investigating whether the resetting of circadian gene expression is a cause or consequence of changes in metabolic phenotypes with time‐restricted diet access.

Studies using HFD feeding and genetic mouse models of obesity suggest that meal‐associated parameters (e.g., size, frequency) are more closely correlated to weight gain than absolute caloric intake [18, 19, 20, 21, 22]. The transition from chow to ad libitum HFD increases meal frequency and size, particularly during the light cycle [3]. The present studies recapitulate these phenotypes in mice given daytime access to HFD, which results in the intake of larger and more frequent HFD meals with no change in chow meal patterns. Similarly, mice given access to HFD during the dark cycle also increased meal size and frequency; surprisingly, this was from chow‐derived meals. HFD meals were actually smaller in mass than chow meals. These findings suggest that mechanisms regulating satiation (meal size) and satiety (meal frequency) can be modulated by both cycle and diet. The presence of the HFD during the daytime impairs satiety and satiation from HFD but not chow, and the opposite occurs when the HFD is available during the nighttime (satiation and satiety is impaired for chow but not HFD). Thus, cycle‐restricted access to HFD influences various feeding behaviors, such as meal patterns, that can ultimately affect the degree of weight gain.

Meal patterns are regulated by mechanosensitive and chemosensitive factors that gauge the mass, caloric content, and composition of a meal [23]. Ad libitum access to HFD decreases meal size by mass but not sufficiently to prevent an increase in meal size by energy content [3]. Thus, mass adjustments are made upon sensing the caloric content of the diet, albeit insufficiently, to avoid a significant increase in energy intake. Contrasting our findings with ad libitum HFD, access to HFD during the light cycle led to increased meal size from the HFD in mass and energy content, suggesting a failure in mechanisms to lower meal mass to account for the increased caloric content. Results from mice given access to HFD during the dark cycle are interesting because the mass of HFD‐derived meals was sufficiently decreased to prevent a significant increase in meal energy content. This suggests that homeostatic mechanisms regulating meal size are intact. However, meal size by mass and energy content from the chow diet was increased in these mice, suggesting a specific dysregulation of satiation mechanisms for the chow diet. This could be secondary to an impairment in homeostatic mechanisms in response to HFD that promote more robust compensatory responses in chow intake. Thus, in mice given access to HFD during the light cycle, the increase in HFD meal size should have been compensated by a decrease in chow meal size. Similarly, in mice given access to HFD during the dark cycle, the presence of the HFD should have resulted in a decrease or maintenance of the size of chow meals. Even short‐term exposure to HFD promotes cellular stress and inflammatory markers [24, 25, 26] in the gut and feeding centers of the brain that could consequently influence feeding behaviors such as meal size. Exposure to HFD can also impair vagal afferent circadian rhythms, which could influence the relaying of meal‐derived information from the gut to the brain [27]. Interestingly, this circadian dysregulation can be ameliorated by time‐restricted feeding [28].

The most striking finding from the present studies is that limiting HFD access to the dark cycle increases intake of and preference for chow. Introducing a novel diet during the period of highest consumption (dark cycle) could result in neophobia. We would anticipate neophobia to diminish over time, and the expected preference for the HFD over the chow diet would eventually emerge. On the contrary, the preference for the chow diet gradually increases over time. This suggests that the hedonic drive to consume palatable substances is context dependent. Supporting this, mice increase their consumption of the HFD when it is freely available, but even a simple task such as requiring a nose poke to obtain food reduces HFD intake (although the nose poke requirement also reduces intake of low‐fat diets) [29]. Circadian mechanisms that regulate feeding may be primed to provide greater homeostatic control relative to hedonic control of intake during the dark cycle compared to the light cycle. As such, the inactive period could be characterized as being more susceptible to dysregulation and a detrimental shift in dietary preferences in the presence of obesogenic substances. Indeed, studies in shift workers show that night shift workers increase their consumption of sugar‐ and fat‐rich, calorie dense foods [9].

Weight gain can also be influenced by changes in EE. Providing mice with ad libitum access to HFD increases EE but not sufficiently to offset the increased energy intake [3]. The same phenomenon was observed here in mice given access to HFD during the light cycle. There was also a slight increase in activity in these mice, which could contribute to the increased EE. Giving mice access to HFD only during the dark cycle had no influence on EE, so the relative protection from weight and fat mass gain in mice fed HFD during the nighttime is not due to increased EE. One possibility is an effect on substrate oxidation, as measured by RER. Daily RER patterns are primarily driven by feeding patterns and the macronutrient composition of the diet. Thus, chow‐fed mice typically display low daytime RER indicative of lipid oxidation and high nighttime RER indicative of increased carbohydrate oxidation. This is because chow‐fed mice eat relatively little during the light cycle and primarily consume the high‐carbohydrate chow at night. Mice given access to HFD during the dark cycle displayed this circadian pattern of RER typical of chow‐fed mice—low daytime RER and increased nighttime RER. This is consistent with their relatively low consumption of food during the day and increased nighttime consumption of chow. Surprisingly, mice given access to HFD during the day displayed a dramatic decrease in RER during the dark cycle, indicative of increased nighttime fat oxidation. This could reflect an attempt to utilize fat resulting from the increased consumption of the HFD during the light cycle. In addition to this, mice exposed to HFD during the day did not display the typical increase in chow intake at night, further contributing to decreasing RER. This increased reliance on fat oxidation was clearly not sufficient to prevent the relative increase in fat mass in mice fed HFD during the light cycle compared to mice fed HFD during the dark cycle. Interestingly, energy balance was equally positive in both groups, so other factors are likely influenced by the timing of HFD access. For example, it is possible that fat absorption is more efficient during the light cycle relative to the dark cycle. Future studies require more comprehensive analyses of factors that contribute to the difference in weight gain and adiposity observed here.

In conclusion, these studies demonstrate that cycle‐restricted access to HFD produces distinct behavioral and body weight outcomes similar to the behaviors and metabolic outcomes observed in humans with disrupted circadian cycles. These findings merit future studies investigating the mechanisms that differentially regulate feeding behavior under these different dietary access conditions.

These studies used male mice, so studies should be conducted in female mice since sex influences behavioral and metabolic effects of HFD. The effect of cycle‐restricted access to other HFD, such as the 45% “Western diet,” should also be investigated. The chow diet used in these studies was used for continuity with our previous studies [3]. However, this chow diet is not a matched low‐fat diet to the HFD, so the nutrient composition (e.g., sucrose content) is not matched between diets. Metabolic measurements were conducted in single‐housed mice, and this invokes a contribution of social isolation and thermal stress.

## Author Contributions

Conceptualization: P.A.F. and J.E.A. Methodology: P.A.F., M.B.B., and J.E.A. Analysis: P.S. and J.E.A. Writing: J.E.A. Funding: J.E.A. All authors reviewed and approved the final version of this manuscript.

## Conflicts of Interest

The authors declare no conflicts of interest.

## Supporting information


**Figure S1:** Daily food and energy intake in light cycle HFD access (LHA) and dark cycle HFD access (DHA) mice. Light cycle data are shaded yellow, dark cycle data are shaded gray, and 24‐h data are shaded white. Daily food intake during the light cycle (A), dark cycle (B), and 24 h (C). Daily energy intake during the light cycle (D), dark cycle (E), and 24 h (F). Daily food intake by diet during the light cycle (G), dark cycle (H), and 24 h (I). Daily energy intake by diet during the light cycle (J), dark cycle (K), and 24 h (L). Cumulative energy intake over the ~4‐week chow and HFD period stratified by chow (dotted bars) and HFD (solid bars). Data are shown as mean ± SEM for *N* = 8 per group and were statistically analyzed using one‐way ANOVA with Tukey's multiple comparison tests. *p* value definitions: **< 0.01, ****< 0.00001.

**Figure S2:** Daily EE, activity, energy balance and RER in light cycle HFD access (LHA) and dark cycle HFD access (DHA) mice. Light cycle data are shaded yellow, dark cycle data are shaded gray, and 24‐h data are shaded white. Daily EE during the light cycle (A), dark cycle (B), and 24 h (C). Daily locomotor activity during the light cycle (D), dark cycle (E), and 24 h (F). Daily energy balance during the light cycle (G), dark cycle (H), and 24 h (I). Daily RER during the light cycle (J), dark cycle (K), and 24 h (L). Data are shown as mean ± SEM for *N* = 8 per group.

**Figure S3:** Daily meal patterns in light cycle HFD access (LHA) and dark cycle HFD access (DHA) mice. Light cycle data are shaded yellow, dark cycle data are shaded gray, and 24‐h data are shaded white. Daily meal size by mass during the light cycle (A), dark cycle (B), and 24 h (C). Daily meal size by energy during the light cycle (D), dark cycle (E), and 24 h (F). Daily meal number during the light cycle (G), dark cycle (H), and 24 h (I). Daily meal duration during the light cycle (J), dark cycle (K), and 24 h (L). Daily intermeal interval during the light cycle (M), dark cycle (N), and 24 h (O). Data are shown as mean ± SEM for *N* = 8 per group.

**Figure S4:** Daily meal patterns by diet in light cycle HFD access (LHA) and dark cycle HFD access (DHA) mice. Light cycle data are shaded yellow, dark cycle data are shaded gray, and 24‐h data are shaded white. Daily meal size by mass and by diet during the light cycle (A), dark cycle (B), and 24 h (C). Daily meal size by energy and by diet during the light cycle (D), dark cycle (E), and 24 h (F). Daily meal number by diet during the light cycle (G), dark cycle (H), and 24 h (I). Daily meal duration by diet during the light cycle (J), dark cycle (K), and 24 h (L). Daily intermeal interval by diet during the light cycle (M), dark cycle (N), and 24 h (O). Data are shown as mean ± SEM for *N* = 8 per group.

## Data Availability

The data that support the findings of this study are available from the corresponding author upon reasonable request.
