# Supplementary figures and images for: Time‐Restricted Access to High‐Fat Diet Influences Weight Gain, Meal Patterns, and Food Preference
Source: Obesity (Silver Spring). 2025 Sep 25;33(12):2304–16. doi: 10.1002/oby.70030 (PMC12636058; doi:10.1002/oby.70030)

## Supplementary Figure 1

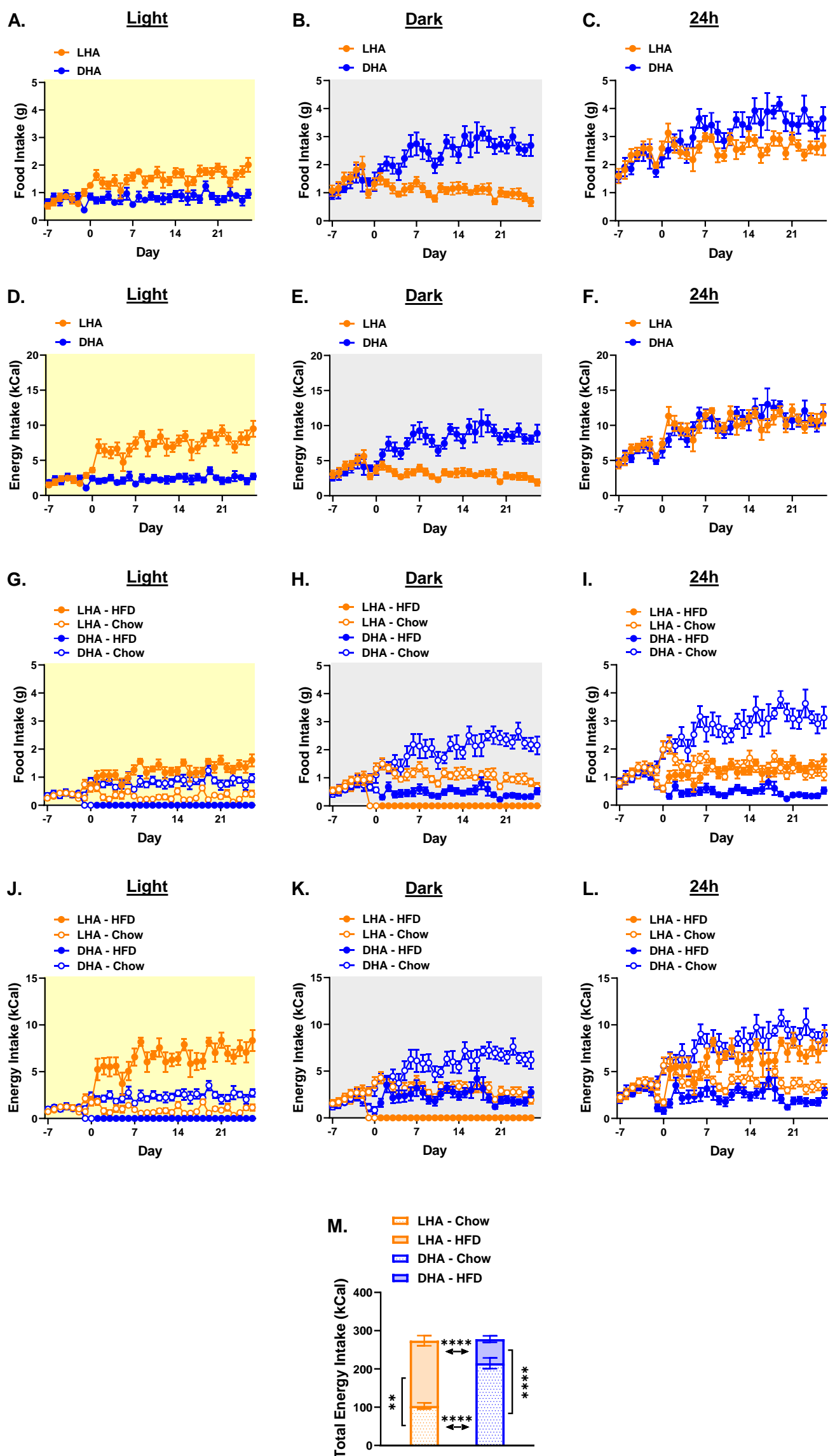

Supplement: Supplementary file 1 — Figure S1: Daily food and energy intake in light cycle HFD access (LHA) and dark cycle HFD access (DHA) mice. Light cycle data are shaded yellow, dark cycle data are shaded gray, and 24‐h data are shaded white. Daily food intake during the light cycle (A), dark cycle (B), and 24 h (C). Daily energy intake during the light cycle (D), dark cycle (E), and 24 h (F). Daily food intake by diet during the light cycle (G), dark cycle (H), and 24 h (I). Daily energy intake by diet during the light cycle (J), dark cycle (K), and 24 h (L). Cumulative energy intake over the ~4‐week chow and HFD period stratified by chow (dotted bars) and HFD (solid bars). Data are shown as mean ± SEM for N = 8 per group and were statistically analyzed using one‐way ANOVA with Tukey's multiple comparison tests. p value definitions: **< 0.01, ****< 0.00001. [file OBY-33-2304-s001.pdf]

# Supplementary Figure 2

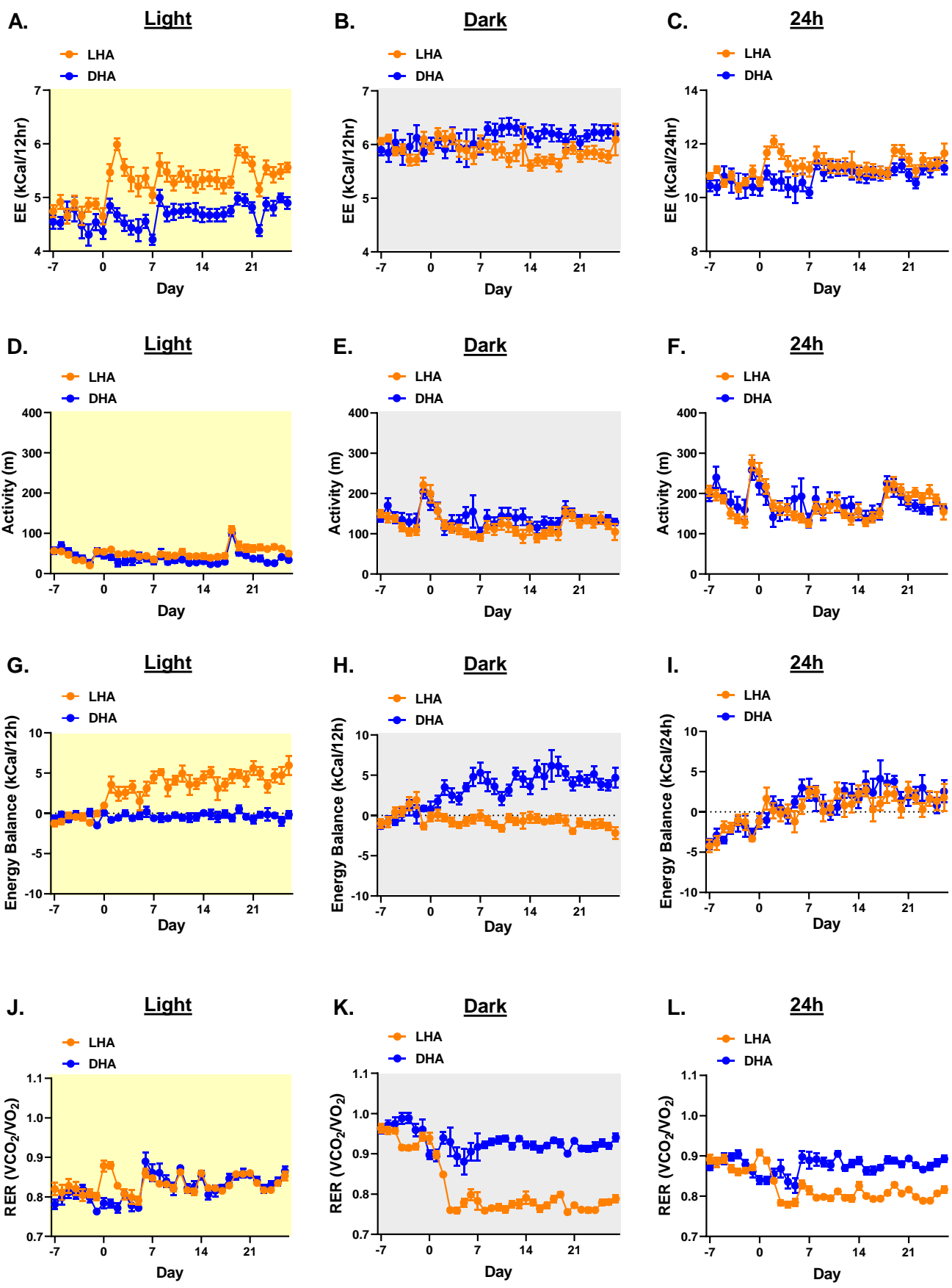

Supplement: Supplementary file 2 — Figure S2: Daily EE, activity, energy balance and RER in light cycle HFD access (LHA) and dark cycle HFD access (DHA) mice. Light cycle data are shaded yellow, dark cycle data are shaded gray, and 24‐h data are shaded white. Daily EE during the light cycle (A), dark cycle (B), and 24 h (C). Daily locomotor activity during the light cycle (D), dark cycle (E), and 24 h (F). Daily energy balance during the light cycle (G), dark cycle (H), and 24 h (I). Daily RER during the light cycle (J), dark cycle (K), and 24 h (L). Data are shown as mean ± SEM for N = 8 per group. [file OBY-33-2304-s004.pdf]

# Supplementary Figure 3

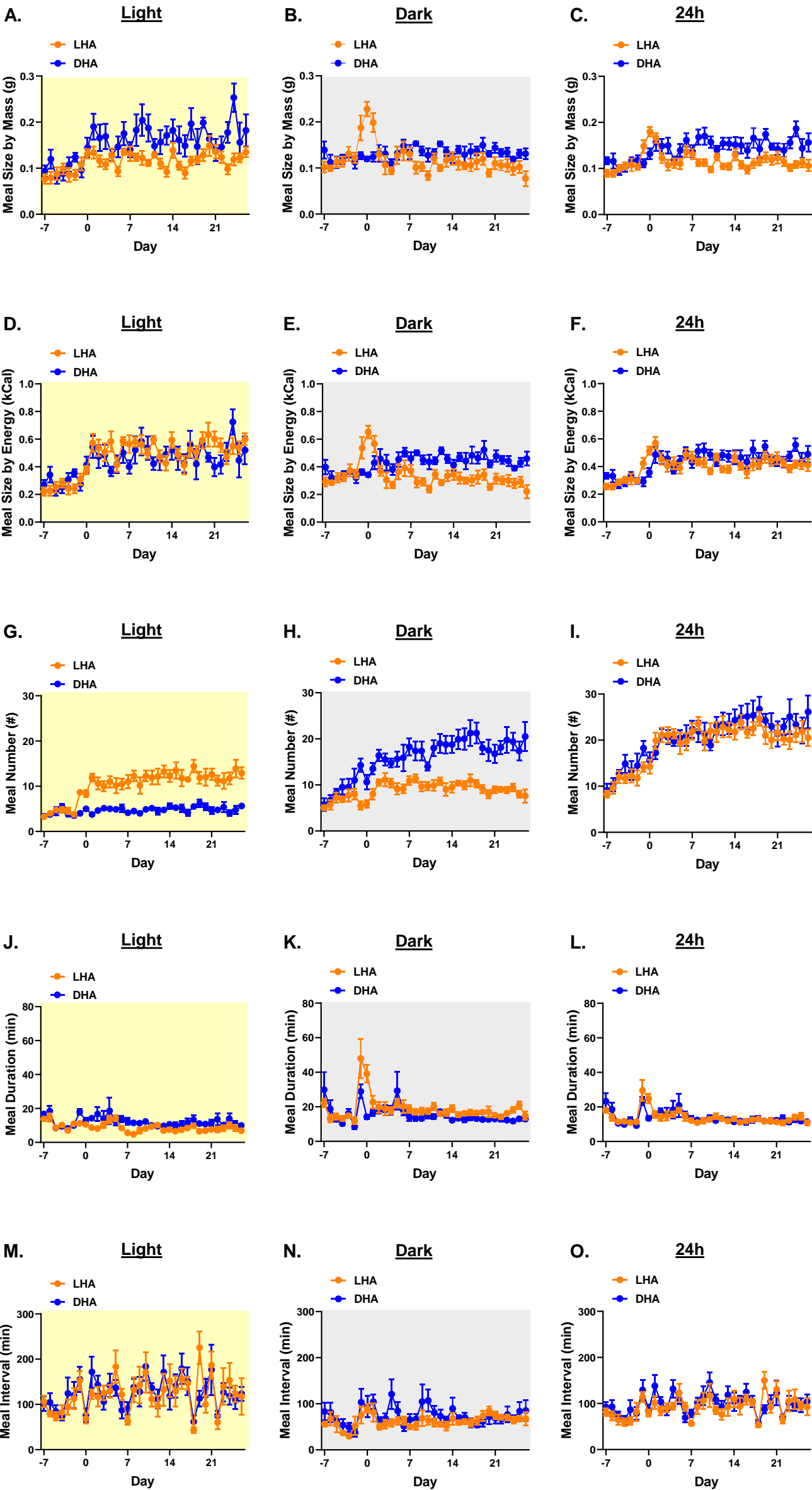

Supplement: Supplementary file 3 — Figure S3: Daily meal patterns in light cycle HFD access (LHA) and dark cycle HFD access (DHA) mice. Light cycle data are shaded yellow, dark cycle data are shaded gray, and 24‐h data are shaded white. Daily meal size by mass during the light cycle (A), dark cycle (B), and 24 h (C). Daily meal size by energy during the light cycle (D), dark cycle (E), and 24 h (F). Daily meal number during the light cycle (G), dark cycle (H), and 24 h (I). Daily meal duration during the light cycle (J), dark cycle (K), and 24 h (L). Daily intermeal interval during the light cycle (M), dark cycle (N), and 24 h (O). Data are shown as mean ± SEM for N = 8 per group. [file OBY-33-2304-s003.pdf]

Supplementary Figure 4

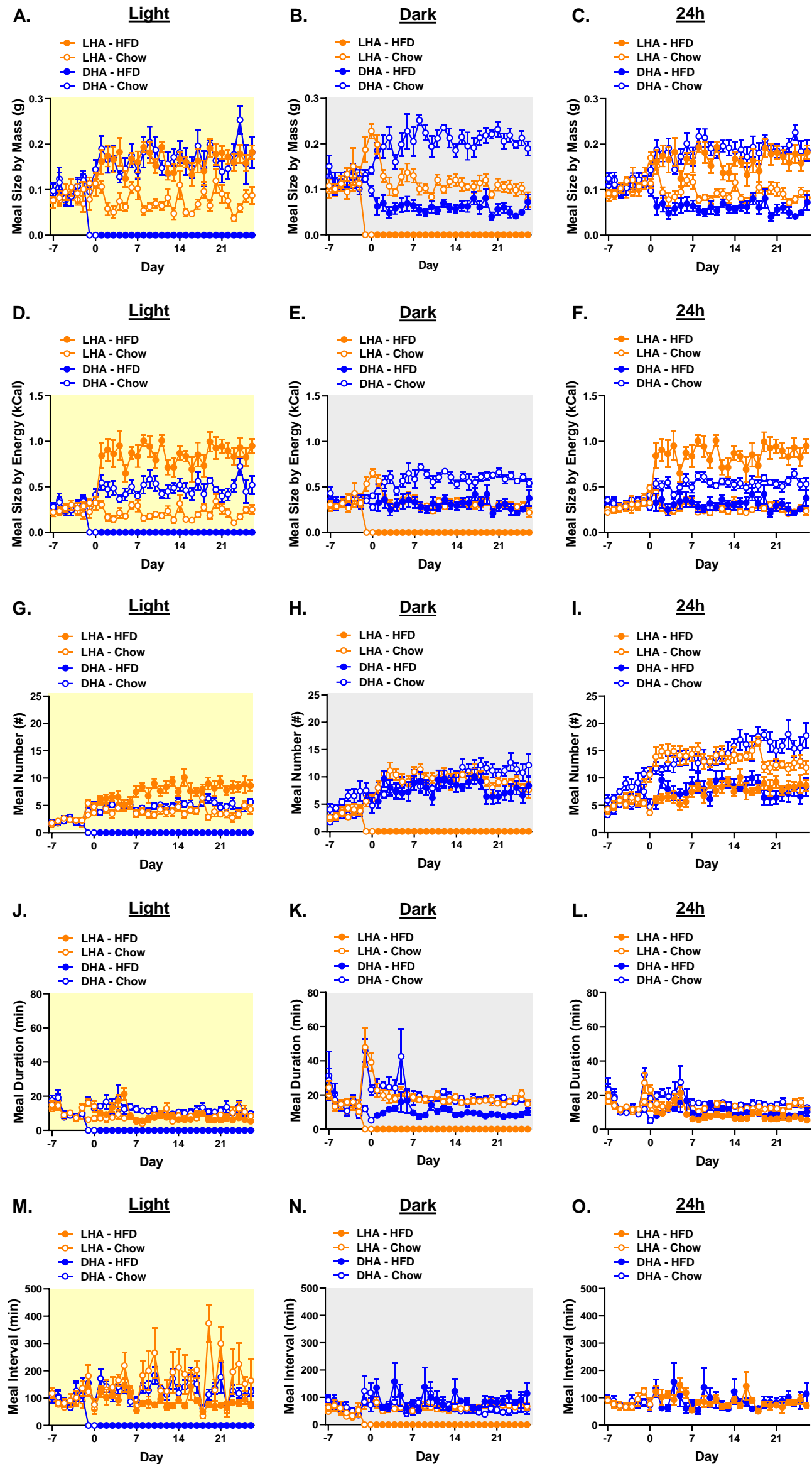

Supplement: Supplementary file 4 — Figure S4: Daily meal patterns by diet in light cycle HFD access (LHA) and dark cycle HFD access (DHA) mice. Light cycle data are shaded yellow, dark cycle data are shaded gray, and 24‐h data are shaded white. Daily meal size by mass and by diet during the light cycle (A), dark cycle (B), and 24 h (C). Daily meal size by energy and by diet during the light cycle (D), dark cycle (E), and 24 h (F). Daily meal number by diet during the light cycle (G), dark cycle (H), and 24 h (I). Daily meal duration by diet during the light cycle (J), dark cycle (K), and 24 h (L). Daily intermeal interval by diet during the light cycle (M), dark cycle (N), and 24 h (O). Data are shown as mean ± SEM for N = 8 per group. [file OBY-33-2304-s002.pdf]
